# Less is More: Modern Neonatology

**DOI:** 10.5041/RMMJ.10344

**Published:** 2018-07-30

**Authors:** Amir Kugelman, Liron Borenstein-Levin, Huda Jubran, Gil Dinur, Shlomit Ben-David, Elena Segal, Julie Haddad, Fanny Timstut, Iris Stein, Imad R. Makhoul, Ori Hochwald

**Affiliations:** 1 Department of Neonatology, Rambam Health Care Campus, Haifa, Israel; 2 The Ruth & Bruce Rappaport Faculty of Medicine, Technion–Israel Institute of Technology, Haifa, Israel

**Keywords:** Iatrogenesis, non-invasive ventilation, premature infants

## Abstract

Iatrogenesis is more common in neonatal intensive care units (NICUs) because the infants are vulnerable and exposed to prolonged intensive care. Sixty percent of extremely low-birth-weight infants are exposed to iatrogenesis. The risk factors for iatrogenesis in NICUs include prematurity, mechanical or non-invasive ventilation, central lines, and prolonged length of stay. This led to the notion that “less is more.” In the delivery room delayed cord clamping is recommended for term and preterm infants, and suction for the airways in newborns with meconium-stained fluid is not performed anymore. As a symbol for a less aggressive attitude we use the term neonatal stabilization rather than resuscitation. Lower levels of oxygen saturations are accepted as normal during the first 10 minutes of life, and if respiratory assistance is needed, we no longer use 100% oxygen but 0.21–0.3 FiO_2_, depending on gestational age and the level of oxygen saturation. We try to avoid endotracheal ventilation by using non-invasive respiratory support and administering continuous positive airway pressure early on, starting in the delivery room. If surfactant is needed, non-invasive methods of surfactant administration are utilized. Use of central lines is shortened, and early feeding of human milk is the routine. Permissive hypercapnia is allowed, and continuous non-invasive monitoring not only of the O_2_ but also of CO_2_ is warranted. “Kangaroo care” and Newborn Individualized Developmental Care and Assessment Program (NIDCAP) together with a calm atmosphere with parental involvement are encouraged. Whether “less is more,” or not enough, is to be seen in future studies.

## INTRODUCTION

Iatrogenesis is any adverse condition caused inadvertently by a medical team as the result of a diagnostic treatment or procedure.^1^ Iatrogenesis has a significant impact on health-care systems.^2^ Our interest is focused on infants in the neonatal intensive care (NICU), which are at high risk for iatrogenesis.^3^,^4^ Ten percent of newborns are born prematurely, and ~1.0% are born <1,500 g.^5^ These infants are exposed to invasive therapies for a prolonged period of hospitalization. Prematurity is associated with retinopathy of prematurity (ROP) and bronchopulmonary dysplasia (BPD), which are also affected by the treatment itself. However, some iatrogenic events (IEs) are not related to prematurity. These IEs are defined as any adverse event causing injury or with a potential for injury (“near miss”) and are related to diagnostic procedures or treatments by the medical team.

**Figure 1:**
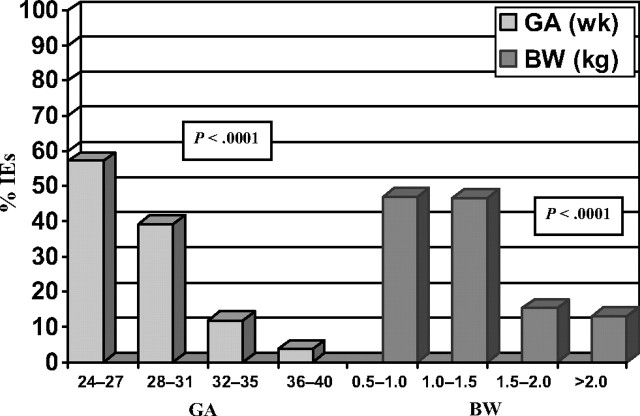
Proportions of Infants with IEs in Gestational Age (GA) and Birth Weight (BW) Strata Reproduced with permission from Pediatrics, Vol. 122, 550–555, Copyright © 2008 by the American Academy of Pediatrics.

We performed a prospective, observational, interventional, multicenter study that included all infants hospitalized in four NICUs in Israel.^3^ Our study revealed an IE prevalence rate of 18.8 infants per 100 hospitalized infants and 0.4 IEs per patient. Sharek and co-workers^6^,^7^ reported on 749 randomly selected charts from 15 NICUs and found 0.74 IEs per patient. Iatrogenesis could be life-threatening (7.9%), significant (40.6%), and harmful (45.1%).^3^ In comparison, the voluntary, anonymous, internet-based reporting system for medical errors in NICUs reported actual harm in 27% of reported IEs.^8^ We considered 83% of the IEs as preventable, whereas Sharek et al.^6^ believed that 56% of IEs are preventable. The most premature infants are more susceptible to intensive, invasive, and prolonged treatments. Thus, they experience more iatrogenesis (Figure 1).^3^,^7^,^9^

We further assessed factors associated with IEs in NICUs.^10^ Univariate analysis indicated that the following infant characteristics were significantly (*P*<0.001) associated with IEs: gestational age, birth weight, severity of initial illness as assessed by the Score for Neonatal Acute Physiology and Perinatal Extension (SNAPPE II), and length of stay (LOS). All four factors demonstrated a significant (*P*<0.001) dose-response relationship with IEs. A prospective study in three NICUs revealed an increased risk for medication errors in more intensive levels of care.^11^

While IEs were associated with LOS on multivariable analysis, a causal relationship could not be established, and it is possible that the IEs were the cause and not the result of prolonged hospitalizations.^12^,^13^ Thus, we cannot claim that LOS is a risk factor for IEs. We can only speculate that shortening the hospitalization would decrease the rate of IEs.

Each category (medications, respiratory or gastrointestinal system, catheterization or other) requires specific interventions, a variety of which are offered in the literature.^14^–^21^ Ligi et al.^22^ assessed the impact of continuous incident reporting and subsequent prevention strategies on the incidence of severe IEs and targeted priorities in admitted neonates. They concluded that such an intervention could improve the quality of care and patient safety.

Parental awareness of IEs might help them to identify IEs early on. The American Academy of Pediatrics encourages the active involvement of parents to prevent errors in both the ambulatory and the inpatient settings.^7^,^14^

The conclusion drawn from these alarming data is that we should be less aggressive and more gentle with premature infants. Our hypothesis is that “less is more,” with adequate precautions and continuous ongoing assessment, making sure that it is enough.

We will discuss some of the recent trends in modern neonatology, implementing and allowing a less intensive/invasive approach.

## THE DELIVERY ROOM

### Prenatal Corticosteroids

Neonatal stabilization starts antenatally by the administration of prenatal corticosteroids. For women at risk of preterm birth, the Cochrane Review^23^ supports using a single course of antenatal corticosteroids to accelerate fetal lung maturation. Antenatal corticosteroid treatment (compared with placebo or no treatment) is associated with a reduction in perinatal and neonatal death and in serious adverse outcomes related to prematurity (Table 1). The rate of BPD is not affected.

**Table 1:** Effects of Prenatal Corticosteroids Given to Women at Risk of Imminent Premature Delivery (based on data of Roberts et al.^23^) and Imminent Late-premature (≥34 weeks’ gestation) Delivery (based on data of Saccone and Berghella^24^)

Outcomes	RR [95% CI]
Women at risk of preterm birth^23^*	
Perinatal death	0.72 [0.58 to 0.89]
Neonatal death	0.69 [0.59 to 0.81]
Respiratory distress syndrome	0.66 [0.56 to 0.77]
Intraventricular hemorrhage	0.55 [0.40 to 0.76]
Necrotizing enterocolitis	0.50 [0.32 to 0.78]
Need for mechanical ventilation	0.68 [0.56 to 0.84]
Systemic infections first 48 hours of life	0.60 [0.41 to 0.88]
Bronchopulmonary dysplasia	0.86 [0.42 to 1.79]
Chorioamnionitis	0.83 [0.66 to 1.06]
Antenatal steroids use in infants ≥34 weeks^24^†	
Respiratory distress syndrome	0.74 [0.61 to 0.91]
Transient tachypnea of newborn	0.56 [0.37 to 0.86]
Use of mechanical ventilation	0.52 [0.36 to 0.76]
Neonatal hypoglycemia	1.61 [1.38 to 1.87]
Length of stay in neonatal intensive care unit	−7.64 days [−7.65 to −7.64]

* Overall: 30 studies (7,774 women and 8,158 infants).

† Overall: 6 randomized control trials (5,698 singleton pregnancies).

These findings are also correct for infants on the border of viability. Antenatal exposure of infants born at 23–24 weeks’ gestation to corticosteroids compared with no exposure was associated with lower mortality and morbidity at most gestations. The effect of antenatal corticosteroids on mortality seems to be larger in infants born at the lowest gestations.^25^

Still debatable is antenatal steroids use at, or after, 34 weeks. This was evaluated in a recent meta-analysis where antenatal corticosteroids were administered to reduce neonatal respiratory morbidity (Table 1).^24^ The study concluded that antenatal steroids at ≥34 weeks’ gestation reduced neonatal respiratory morbidity.^24^ Finally, a recent American College of Obstetricians and Gynecologists (ACOG) recommendation^26^ advises that corticosteroid administration before anticipated preterm birth is one of the most important antenatal therapies available to improve newborn outcomes. For pregnant women at risk of a preterm delivery within 7 days, at 24.0–33.6 weeks’ gestation, a single course of corticosteroids is recommended; it may also be considered for pregnant women starting at 23.0 weeks’ gestation, based on the family’s resuscitation decision. Betamethasone administration may be considered in pregnant women at 34.0–36.6 weeks’ gestation who are at risk of preterm birth within 7 days, if they have not received a previous course of antenatal corticosteroids.

### Delayed Cord Clamping (DCC)

The ACOG recommends delaying umbilical cord clamping in vigorous term and preterm infants for at least 30–60 seconds after birth.^27^ There is a small increase in the incidence of jaundice that requires phototherapy in term infants undergoing delayed umbilical cord clamping (DCC). Delayed umbilical cord clamping does not increase the risk of postpartum hemorrhage. In term infants, DCC increases hemoglobin levels at birth and improves iron stores in the first several months of life, which may have a favorable effect on developmental outcomes. In preterm infants, DCC is associated with improved transitional circulation, better establishment of red blood cell volume, decreased need for blood transfusion, and a lower incidence of necrotizing enterocolitis (NEC) and intraventricular hemorrhage (IVH).

A large randomized controlled trial (RCT) found that, among preterm infants delivered before 30 weeks’ gestational age, DCC did not reduce the incidence of the combined outcomes of death or major morbidity (defined as severe brain injury on postnatal ultrasonography, severe retinopathy of prematurity, NEC, or late-onset sepsis) at 36 weeks’ gestation as compared to immediate cord clamping.^28^ In the most recent review^29^ DCC reduced hospital mortality in premature infants and in a subgroup of infants born at ≤28 weeks’ gestation (Table 2). Delayed cord clamping increased peak hematocrit and reduced the proportion of infants needing blood transfusion. However, DCC did not reduce the incidence of intubation for resuscitation, mechanical ventilation, IVH, brain injury, BPD, patent ductus arteriosus, NEC, late-onset sepsis, or ROP (Table 2). Potential harms of DCC included polycythemia and hyperbilirubinemia. The review by Fogarty et al. provided clear evidence that DCC reduced hospital mortality, supporting the ACOG guidelines.^29^

**Table 2:** Outcomes of Delayed Cord Clamping (DCC) versus Early Cord Clamping Based on Data of Fogarty et al.^29^

Outcome	RR [95% CI]
Hospital mortality in premature infants*	0.68 [0.52 to 0.90]
Hospital mortality in infants ≤28 weeks’ gestation†	0.70 [0.51 to 0.95]
Cardiorespiratory support at resuscitation	0.89 [0.71 to 1.11]
Intubation in delivery room	0.96 [0.82 to 1.13]
Severe intraventricular hemorrhage	0.87 [0.59 to 1.27]
Periventricular leukomalacia	0.71 [0.39 to 1.27]
Mechanical ventilation	0.95 [0.84 to 1.07]
Chronic lung disease ≥36 weeks	1.02 [0.93 to 1.12]
Necrotizing enterocolitis	0.88 [0.65 to 1.18]
Late-onset sepsis	0.95 [0.80 to 1.13]
Severe retinopathy of prematurity	0.74 [0.51 to 1.07]
Peak hematocrit, %	MD: 2.73 [1.94 to 3.52]
Blood transfusion	0.81 [0.74 to 0.87]
Polycythemia (hematocrit >65%)	2.65 [1.61 to 4.37]
Peak bilirubin, μmol/L	MD: 4.43 [1.15 to 7.71]

* Including 2,834 premature infants in 18 RCTs.

† Including 996 infants ≤28 weeks’ gestation in 3 RCTs.

MD, mean difference.

### Oxygenation

Since the optimal concentration of oxygen (FiO_2_) required for stabilization of the newly born infant had not yet been established, Kamlin et al.^30^ wanted to determine the range of preductal peripheral capillary oxygen saturation (SpO_2_) in the first minutes of life in healthy newborn infants. They found that the median (interquartile range) SpO_2_ at 1 minute was 63% (53%–68%) with a gradual rise in SpO_2_ over time, and a median SpO_2_ at 5 minutes of 90% (79%–91%).

With the knowledge that oxygen can be toxic, the 2010 International Consensus for Neonatal resuscitation (NRP)^31^ came out with the following recommendations. For term infants resuscitated at birth with positive-pressure ventilation, it is best to begin with air rather than 100% oxygen. If the heart rate does not increase or oxygenation (guided by oximetry) remains unacceptable, despite effective ventilation, a higher oxygen concentration should be considered. Since many preterm babies under 32 weeks’ gestation cannot achieve target saturations in air, an oxygen–air blend may be judiciously administered, and ideally guided by pulse oximetry. Both hyperoxemia and hypoxemia should be avoided. If an oxygen–air blend is unavailable, resuscitation should be initiated with air.

### Airway Suctioning

The 2010 NRP guidelines^31^ made another change of practice, again in the direction of less activism. They no longer recommend routine intrapartum oropharyngeal and nasopharyngeal suctioning for infants born with clear or meconium-stained amniotic fluid. Regarding tracheal suctioning, no evidence is available to support or refute routine endotracheal suctioning of depressed infants born through meconium-stained amniotic fluid.

## THE NEONATAL INTENSIVE CARE UNIT (NICU)

### Non-invasive Respiratory Support

Preterm infants with respiratory distress syndrome (RDS) have low pulmonary compliance and high thoracic-cage compliance. Thus, to avoid loss of lung volume and atelectasis they need gentle support of functional residual capacity (FRC) and surfactant replacement. The aim of the respiratory treatment of RDS is to oxygenate and ventilate the premature infants using these two elements of treatment while preventing death, BPD, and neurological morbidity.^32^ Bronchopulmonary dysplasia has a complex pathophysiology, based on arrest of maturation and multi-heat phenomena.^33^ Thus, there is no magic bullet, and the prevention of BPD warrants the implementation of a comprehensive approach.^34^

Even few breaths by positive pressure ventilation were found to be harmful to the lungs.^35^,^36^ Furthermore, endotracheal ventilation was found to be associated with cerebral palsy and low mental developmental index (MDI).^37^ Thus, to achieve the goals of respiratory support we try to avoid endotracheal ventilation.

If endotracheal ventilation is needed in premature infants with RDS, there is no preference of conventional ventilation or high-frequency ventilation when assessing death or BPD or severe adverse neurological outcomes.^38^ If conventional ventilation is used, volume-targeted ventilation is preferred over pressure-limited ventilation in order to reduce death or BPD, pneumothoraxes, hypocarbia, severe cranial ultrasound pathologies, and duration of ventilation.^39^ However, innovative modes of ventilation and the use of surfactant did not reduce substantially the incidence of BPD. The rate of BPD was found to correlate with the use and length of endotracheal mechanical ventilation.^40^ Furthermore, BPD in itself is associated with adverse neurodevelopmental outcome.^41^

Allowing nasal respiratory support as a safe and efficient alternative to endotracheal ventilation and surfactant in the most premature infants was the result of a thorough evaluation by two large RCTs. Morley et al.^42^ randomly assigned 610 infants who were born at 25–28 weeks’ gestation to nasal continuous positive airway pressure (NCPAP) or intubation and ventilation at 5 minutes after birth. They concluded that early NCPAP did not significantly reduce the rate of death or BPD, as compared with intubation. The SUPPORT trial^43^ was a randomized, multicenter trial, involving infants who were born at 24.0–27.6 weeks’ gestation. Infants were randomly assigned to intubation and surfactant treatment (within 1 hour after birth) or to NCPAP treatment initiated in the delivery room. A total of 1,316 infants were enrolled in the study. This study supported consideration of NCPAP as an alternative to intubation and surfactant in preterm infants. The primary outcome (death or BPD) rates did not differ significantly between the two groups. However, infants in the NCPAP group required less frequent intubation or postnatal corticosteroids for BPD (*P*<0.001), required fewer days of mechanical ventilation (*P*=0.03), and were more likely to survive and be free from mechanical ventilation by day 7 (*P*=0.01).

These studies allow the consideration of NCPAP as an alternative to intubation and surfactant in extremely preterm infants and reveal a small but significant benefit in long-term outcomes. A meta-analysis^44^ including these studies and a total of 3,289 infants found that the combined odds ratio (95% confidence interval) of death or BPD was 0.83 (0.71–0.96) using NCPAP versus intubation and surfactant. The number needed to treat was 35 infants. Avoiding endotracheal mechanical ventilation had no influence on the incidence of severe IVH. They concluded that strategies aimed at avoiding endotracheal mechanical ventilation in infants <30 weeks’ gestational age have a small but significant beneficial impact on preventing BPD.

Attempts to enhance NCPAP to achieve a better outcome for nasal respiratory support led to the use of nasal intermittent positive pressure ventilation (NIPPV), defined as a method of augmenting NCPAP by delivering ventilator breaths via nasal prongs. The rationale for using NIPPV is the administration of “sigh” to the infant, thus opening microatelectasis and recruiting more ventilation units. Synchronized NIPPV compared with NCPAP has been found to activate the respiratory drive,^45^ improves thoraco-abdominal synchrony,^46^ stabilizes the chest wall,^46^ improves lung mechanics,^46^ and decreases the breathing effort in premature infants.^47^

In clinical studies, early NIPPV appears to be superior to NCPAP for reducing respiratory failure and the need for endotracheal tube ventilation among preterm infants with RDS.^48^ However, infants randomized to NIPPV have comparable risk of BPD. For the initial therapy of RDS high-flow nasal cannula (HFNC) compared with NCPAP in 564 infants with gestational age ≥28 weeks was associated with significantly higher rates of treatment failure within 72 hours. “Rescue” NCPAP use resulted in similar intubation rates in the two treatment groups.^49^ Kugelman et al. in infants >1,000 g found no difference between HFNC and NIPPV, but that study included only 76 infants.^50^

For post-extubation, a meta-analysis showed that synchronized NIPPV reduces the incidence of extubation failure and the need for re-intubation within 48 hours–1 week more effectively than NCPAP; however, the rate of BPD or mortality was not changed. The number needed to treat was 3 infants.^51^ High-flow nasal cannula post-extubation in 303 infants <32 weeks’ gestation was found to be non-inferior to the use of NCPAP, with treatment failure occurring in 34% of the infants in the nasal-cannula group and in 25% of the infants in the NCPAP group.^52^ Almost half the infants in whom treatment with HFNC failed were successfully treated with NCPAP without re-intubation. The incidence of nasal trauma was significantly lower in the nasal-cannula group than in the CPAP group (*P*=0.01).

### Surfactant Treatment

Surfactant was a major breakthrough in the treatment of premature infants with RDS. A crucial question was whether surfactant should be given as a preventive therapy or only as rescue therapy. This is important if we try to avoid endotracheal ventilation, as surfactant traditionally is given via the endotracheal tube. The Cochrane Review^53^ concluded that recent large trials that reflect current practice (including greater utilization of antenatal steroids and routine post-delivery stabilization on NCPAP) demonstrate less risk of BPD or death when using early stabilization on NCPAP with selective surfactant administration to infants requiring intubation.

While non-invasive ventilation seems to be safe, its success depends on gestational age.^42^,^43^,^54^ There is still a significant role for surfactant in the treatment of RDS, especially in extremely low-birth-weight infants (~50% will need intubation and surfactant). In the last decade, few methods of gentle administration of surfactant were developed to allow the infant to benefit from both, surfactant and nasal respiratory support. The INSURE approach (INtubation SURfactant Extubation; in which surfactant is administered during brief intubation followed by immediate extubation to NCPAP), when compared with later selective surfactant administration, continued mechanical ventilation, and extubation from low respiratory support, was associated with less need for mechanical ventilation, lower incidence of BPD (at 28 days), and fewer air-leak syndromes.^55^ Gopel et al.^56^ showed that the application of surfactant via a thin catheter to spontaneously breathing preterm infants receiving NCPAP reduces the need for mechanical ventilation. This method is called LISA (less invasive surfactant administration) or MIST (minimal invasive surfactant therapy). A recent meta-analysis showed that, among preterm infants, LISA use was associated with the lowest likelihood of the composite outcome of death or BPD at 36 weeks’ postmenstrual age.^57^ A new approach that is under investigation, which will allow avoidance of direct instillation of surfactant to the trachea, would be aerosolization of the surfactant.^58^

### Non-invasive Ventilation and Surfactant Treatment

To summarize, a lot of effort is invested in the non-invasive ventilation approach. The outcomes of that approach in the long run are still to be investigated. A recent study did not find benefits, concluding that despite substantial increases in the use of less invasive ventilation after birth, there was no significant decline in oxygen dependence at 36 weeks and no significant improvement in lung function in childhood over time.^59^ The results of that study could have different interpretations.^60^,^61^ The recent cohorts in that study, 1997 and 2005, showed no significant difference in the rate of endotracheal ventilation. It has been shown that even short exposure to endotracheal positive pressure ventilation is harmful.^35^,^36^ Thus, it is possible to conclude from the study of Doyle et al.^59^ that every effort should be made to minimize the use of endotracheal ventilation by using more non-invasive ventilation. At the same time, there was a striking decrease in the use of postnatal glucocorticoids between these cohorts, from 46% in 1997 to 23% in 2005. The differences between these periods could explain the surprising results found by Doyle et al.^59^ To overcome the possible effect of the decreased use of glucocorticoids, it is possible that we should consider other policies of using postnatal glucocorticoids that do not adversely affect the neurodevelopmental outcome. These could include inhaled glucocorticoids,^62^ low-dose hydrocortisone,^63^ or intratracheal glucocorticoids with surfactant.^64^

Considering the complex nature of BPD,^33^ a comprehensive approach^34^ will be needed to show its reduction. Beyond understanding the biologic and physiologic rationale of such an approach, studying it in RCTs seems to be an impossible mission with the current knowledge due to ethical constraints.

### Non-invasive CO_2_ Monitoring

The non-invasive approach is seen also in seeking methods of non-invasive continuous monitoring of carbon dioxide (CO_2_) in the NICU. Hypercarbia and hypocarbia are to be avoided in premature infants because of possible neurological and respiratory deleterious effects.^65^–^67^ Carbon dioxide can be monitored by capnography^68^,^69^ and by transcutaneous CO_2_ monitoring (TcCO_2_).^70^

Kugelman et al.^71^ showed that continuous distal end tidal CO_2_ monitoring improved control of CO_2_ levels within a safe range during conventional ventilation in NICUs. The prevalence of IVH or periventricular leukomalacia rate was lower in the monitored group. However, the number of extremely premature infants was small, and these results should be interpreted with caution. Larger studies in the vulnerable population are needed in order to show short- and long-term clinical benefits of continuous CO_2_ monitoring. We should probably perform continuous non-invasive monitoring of ventilation (CO_2_) similarly to what we do for oxygenation by using continuous pulse oximetry. This can be done by capnography and TcCO_2_. Capnography and TcCO_2_ monitoring should be viewed as complementary technologies in various clinical scenarios in the NICU.

### “Kangaroo Care” and Newborn Individualized Developmental Care and Assessment Program (NIDCAP)

“Kangaroo care” and Newborn Individualized Developmental Care and Assessment Program (NIDCAP) are adopted by the modern neonatology.

Kangaroo mother care (KMC) was defined as “skin-to-skin contact between a mother and her newborn, frequent and exclusive or nearly exclusive breastfeeding, and early discharge from hospital.”^72^ A Cochrane Review^72^ aimed to determine whether evidence is available to support the use of KMC in low-birth-weight infants as an alternative to conventional neonatal care before or after the initial period of stabilization with conventional care. Their updated review supports use of KMC in low-birth-weight infants as an alternative to conventional neonatal care, mainly in resource-limited settings.

The immaturity of their organ systems causes preterm infants to experience a range of morbidities. There is concern that a non-gentle or an un-comfortable environment in the NICU may have an unfavorable effect on this morbidity or result in iatrogenesis. Environmental modification can minimize the iatrogenic effects. Developmental care represents a broad category of interventions designed to minimize NICU environmental stress. Such interventions may include control of external stimuli (vestibular, auditory, visual, tactile), clustering of nursery care activities, and positioning or swaddling of the preterm infant. Individual strategies have also been combined to form programs, such as the NIDCAP. A Cochrane Review^73^ looking at the effect of NIDCAP concluded that determination of the effect of any single intervention is difficult because of the inclusion of multiple interventions in most studies. Although the evidence indicates a limited benefit for developmental care interventions overall, and no data suggest harmful effects, there have been a large number of outcomes with debatable effects. For each intervention there is only support by single small studies which were not repeated. The interventions have an economic impact that needs to be considered. A more recent systematic review of preterm infants (*n*=627) found no evidence that NIDCAP improves long-term neurodevelopmental or short-term medical outcomes.^74^ The composite primary outcomes of death or major sensorineural disability at 18 months corrected age or later in childhood (RR 0.89 [95% CI 0.61 to 1.29]) and survival free of disability at 18 months corrected age or later in childhood (RR 0.97 [95% CI 0.69 to 1.35]) were not significantly different between the NIDCAP and control groups.

## SUMMARY

In modern neonatology we tend to treat more infants on the border of viability: born at 24 weeks’ and even 23 weeks’ gestation. This means that the extremely premature infants spend more time in the NICU until maturation and are exposed to invasive treatments including respiratory and nutritional support (central lines), with all the morbidities associated with extreme prematurity.

At the same time, we are aiming for a more gentle approach as discussed previously in all areas of interventions. This alleged contradiction makes the treatments of these infants a huge challenge.

While aiming for a more gentle approach, we should always assess and re-evaluate our treatments and routines, because sometimes “less” might not be enough. For example, keeping lower levels of oxygen saturations in extremely premature infants to prevent ROP or BPD was found to be associated with increased rate of death.^75^ Future studies with short- and long-term follow-up will give guidance on how to implement and improve our care in the face of a changing/evolving reality.

## Abbreviations

ACOG: American College of Obstetricians and Gynecologists
BPD: bronchopulmonary dysplasia
DCC: delayed cord clamping
HFNC: high-flow nasal cannula
IE: iatrogenic event
IVH: intraventricular hemorrhage
KMC: kangaroo mother care
LISA: less invasive surfactant administration
LOS: length of stay
NCPAP: nasal continuous positive airway pressure
NEC: necrotizing enterocolitis
NICU: neonatal intensive care unit
NIDCAP: Newborn Individualized Developmental Care and Assessment Program
NIPPV: nasal intermittent positive pressure ventilation
RCT: randomized controlled trial
RDS: respiratory distress syndrome
ROP: retinopathy of prematurity
SpO_2_: peripheral capillary oxygen saturation
TcCO_2_: transcutaneous CO_2_.
